# α1-antitrypsin promotes SPLUNC1-mediated lung defense against *Pseudomonas aeruginosa* infection in mice

**DOI:** 10.1186/1465-9921-14-122

**Published:** 2013-11-09

**Authors:** Di Jiang, Rebecca Persinger, Qun Wu, Ashley Gross, Hong Wei Chu

**Affiliations:** 1 Department of Medicine, National Jewish Health, Denver, CO, USA; 2 Grifols, Inc., Research Triangle Park, Durham, NC, USA; 3 Department of Medicine, Room A639, National Jewish Health, 1400 Jackson Street, Denver, CO 80206, USA

**Keywords:** SPLUNC1, α1-antitrypsin, *Pseudomonas aeruginosa* infection, Human neutrophil elastase

## Abstract

**Background:**

*Pseudomonas aeruginosa* (PA) infection is involved in various lung diseases such as cystic fibrosis and chronic obstructive pulmonary disease. However, treatment of PA infection is not very effective in part due to antibiotic resistance. α1-antitrypsin (A1AT) has been shown to reduce PA infection in humans and animals, but the underlying mechanisms remain unclear. The goal of our study is to test whether a novel endogenous host defense protein, short palate, lung, and nasal epithelium clone 1 (SPLUNC1), is involved in the therapeutic effect of A1AT during lung PA infection.

**Method:**

SPLUNC1 knockout (KO) and littermate wild-type (WT) mice on the C57BL/6 background were intranasally infected with PA to determine the therapeutic effects of A1AT. A1AT was aerosolized to mice 2 hrs after the PA infection, and mice were sacrificed 24 hrs later. PA load and inflammation were quantified in the lung, and SPLUNC1 protein in bronchoalveolar lavage (BAL) fluid was examined by Western blot.

**Results:**

In WT mice, PA infection significantly increased neutrophil elastase (NE) activity, but reduced SPLUNC1 protein in BAL fluid. Notably, PA-infected mice treated with A1AT versus bovine serum albumin (BSA) demonstrated higher levels of SPLUNC1 protein expression, which are accompanied by lower levels of NE activity, lung bacterial load, and pro-inflammatory cytokine production. To determine whether A1AT therapeutic effects are dependent on SPLUNC1, lung PA load in A1AT- or BSA-treated SPLUNC1 KO mice was examined. Unlike the WT mice, A1AT treatment in SPLUNC1 KO mice had no significant impact on lung PA load and pro-inflammatory cytokine production.

**Conclusion:**

A1AT reduces lung bacterial infection in mice in part by preventing NE-mediated SPLUNC1 degradation.

## Background

Bacterial infection is involved in the pathogenesis of cystic fibrosis (CF) and chronic obstructive pulmonary disease (COPD) [1]. Persistent lung *Pseudomonas aeruginosa* (PA) infection is one the most common causes associated with the exacerbations of CF and COPD [1-3]. One of the major challenges in the treatment of PA infection is the rising antibiotic resistance [4]. A previous study has demonstrated the therapeutic effect of α1-antitrypsin (A1AT) against PA infection in CF patients [5]. Animal studies also suggested the anti-PA effects of A1AT [5,6], but how A1AT improves host defense against PA infection remains unknown.

PA infection in the lung elicits a robust inflammatory response such as recruitment and activation of neutrophils. Neutrophil elastase (NE) is released during lung inflammation, which in turn may cause detrimental effects such as tissue destruction seen in emphysema. We have demonstrated that human NE (HNE) impairs airway epithelial defense functions against bacterial infections by degrading short palate, lung, and nasal epithelium clone 1 (SPLUNC1) protein [7]. SPLUNC1 is a 25-kDa secretory protein from large airway epithelial cells. It has been shown to exert host defense function against several strains of bacteria including PA, non-typeable *Haemophilus influenzae* (NTHi) and *Mycoplasma pneumoniae*[7-9]. However, whether NE *in vivo* degrades SPLUNC1 and subsequently impair lung defense against PA infection has not been investigated.

A1AT is a 52-kDa serine protease inhibitor mainly produced by the liver and in the lung by macrophages and epithelial cells. In the lung, A1AT is a potent inhibitor of neutrophil elastase protecting the lung tissue from proteolytic degradation [10]. In individuals with a genetic deficiency of A1AT there is an imbalance between neutrophil elastase and A1AT in the lung increasing the risk of developing emphysema [11]. Purified human A1AT has been used to treat A1AT deficiency by augmenting A1AT levels and inhibiting NE activity [12,13]. In the current study, we hypothesized that A1AT prevents NE-mediated SPLUNC1 degradation and subsequently enhances lung defense against PA infection. To test our hypothesis, we utilized the PA infection model in wild-type and SPLUNC1 knockout (KO) mice with or without A1AT treatment. First, we confirmed that PA infection reduces SPLUNC1 expression in mouse bronchoalveolar lavage (BAL) fluid. Second, we demonstrated that A1AT increased SPLUNC1 levels in PA-infected wild-type mice, and decreased lung PA load. Third, we found that SLPUNC1 is required to improve lung defense against PA.

## Methods

### Animals

SPLUNC1 knockout (KO) and littermate control wild-type (WT) mice on the C57BL/6 background were used for the current study. SPLUNC1 KO mice were generated as we previously reported [14]. All mice were bred and housed in our biological resource center under pathogen-free conditions, and tested to establish that they were virus and *M. pulmonis* free. All the animal procedures were approved by the IACUC at National Jewish Health.

### *Pseudomonas aeruginosa* culture

*Pseudomonas aeruginosa* (PA) strain PAO1 was kindly provided by Dr. Michael Schurr at the University of Colorado Denver, and was stored at −80°C. For each experiment, bacteria were first streaked onto a Tryptic-Soy agar plate and cultured for 18–22 hrs at 37°C. An individual colony was then inoculated into Tryptic-Soy medium and shaked at 37°C to grow the bacteria until 1 × 10^8^ CFUs/ml were achieved as determined by spectrophotometry (optical density at 600 nm = 0.5).

### Mouse model of PA infection with α1-antitrypsin treatment

PA (1.5 × 10^7^ CFUs/mouse in 30 μl saline) or saline (control) was intranasally inoculated to WT and SPLUNC1 KO mice. After 2 hrs of PA infection, purified human alpha-1 proteinase inhibitor (A1AT or Prolastin-C, Grifols Inc., Research Triangle Park, NC) or bovine serum albumin (BSA, Sigma-Aldrich, as the A1AT control) were aerosolized to mice. To deliver A1AT or BSA, WT and SPLUNC1 KO mice were placed in a Plexiglas chamber and treated with aerosolized A1AT or BSA (0.5 mg/ml, total volume = 10 ml) for 30 minutes by using an ultrasonic nebulizer (De Vilbiss) at an airflow rate of 8 L/min. After 24 hrs of PA infection, lung lavage was performed to collect bronchoalveolar lavage (BAL) fluid for cell count, and measurement of KC and NE activity.

### BAL and lung tissue processing

Mouse lungs were lavaged with 1 ml of sterile saline. Cell-free BAL fluid were stored at −80°C for cytokine analysis and Western blot. Cytospins of BAL cells were stained with a Diff-Quick Kit (IMEB INC., San Marcos, CA), and leukocyte differentials were determined as percentage of 500 counted leukocytes. The left lung lobe from infected mice was homogenized in PBS, the homogenates were then cultured on Tryptic-Soy agar plates to quantify PA levels.

### Western blot analysis of mouse SPLUNC1 protein

Western blot analysis was carried out to quantify SPLUNC1 protein. In brief, 30 μl BAL fluid was electrophoresed on 10% SDS-polyacrylamide gel electrophoresis, transferred onto a nitrocellulose membrane, blocked with the Western blocking buffer, and incubated with a sheep anti-mouse SPLUNC1 antibody (R&D Systems, Inc., Minneapolis, MN) overnight at 4°C. After washes in PBS with 0.1% Tween 20, the membranes were incubated with an anti-sheep IgG conjugated to horseradish peroxidase. Membranes were stained with Ponceau S solution to normalize total protein load by comparing the 68-kDa protein (corresponding to albumin) levels. Densitometry was performed using the NIH Image-J software. The ratio of SPLUNC1/albumin was used to normalize SPLUNC1 protein expression in BAL fluid.

### ELISA of mouse KC

KC, a homolog of human IL-8, in mouse BAL fluid was determined by using a mouse KC DuoSet ELISA Development kit (R&D Systems, Minneapolis, MN) as per manufacturer’s instruction.

### Neutrophil elastase (NE) activity assay in mouse BAL fluid

NE activity in mouse BAL fluid samples was measured using the substrate N-(methoxysuccinyl)-Ala-Ala-Pro-Val p-nitroanilide (Sigma-Aldrich) [15]. The samples were mixed with an equal volume of 0.1 M HEPES buffer containing 0.5 mmol/L NaCl (pH 7.5), and then transferred to a 96-well plate. Saline solution mixed with NaCl/HEPES buffer was used as a negative control. The substrate, 2 mmol/L N-(methoxysuccinyl)-Ala-Ala-Pro-Val p-nitroanilide, was added to each well. After 5 minutes of incubation, liberation of p-nitroaniline was measured at 405 nm using a spectrophotometer. The OD readings were used to reflect NE activity.

### Statistical analysis

Data are presented as means ± SEM. One-way analysis of variance (ANOVA) was used for multiple comparisons, and a Tukey’s post hoc test was applied to illustrate the significant differences between two groups. Student’s *t* test was used when only two groups were compared. A *p* value < 0.05 was considered significant.

## Results

### Reduced SPLUNC1, but increased NE activity in BAL fluid of PA-infected wild-type mice

**Figure 1:**
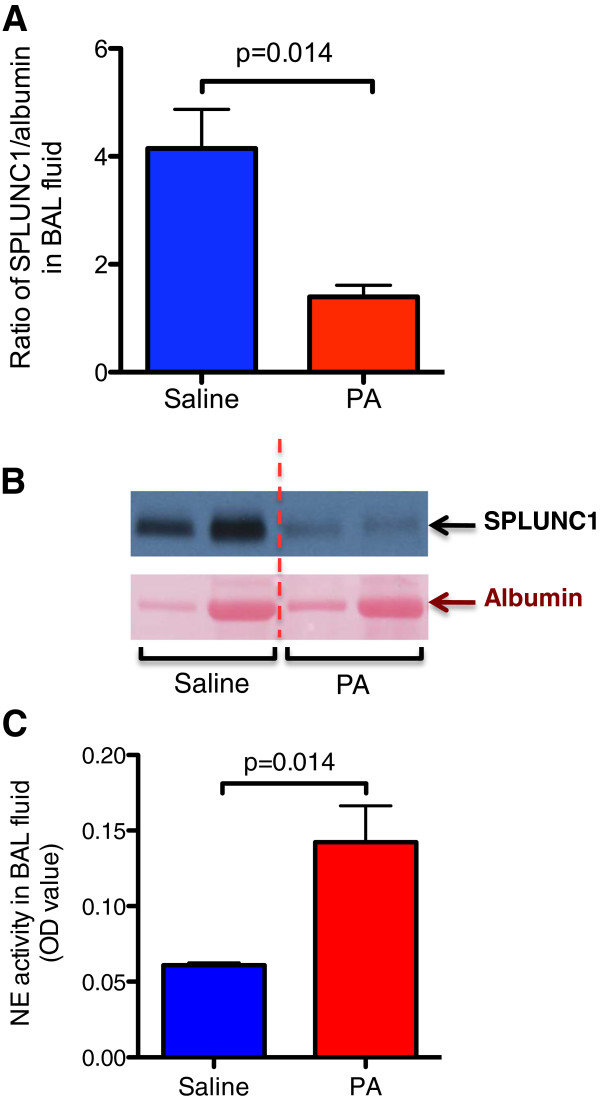
***Pseudomonas aeruginosa *(PA) infection reduces SPLUNC1 and increases neutrophil elastase (NE) activity in bronchoalveolar lavage (BAL) fluid of wild-type (WT) mice.** BAL fluid from WT mice was processed for SPLUNC1 protein Western blot. **(A)** – Quantitative analysis of BAL fluid SPLUNC1 protein expression normalized by albumin. **(B)** – Representative Western blot image of SPLUNC1 and albumin. The vertical dotted red line separates the saline group from the PA infection group. The two lanes under saline or PA treatment represent SPLUNC1 data from two different mice. **(C)** – NE activity was examined by an NE activity assay as described in Materials and methods. N = 4 – 5 mice per group. Data are expressed as means ± SEM.

After 24 hrs of PA infection in wild-type mice, SPLUNC1 protein in BAL fluid was examined by Western blot analysis. As shown in Figure 1A and 1B, PA infection reduced SPLUNC1 protein expression in BAL fluid. In contrast to the SPLUNC1 data, NE activity was significantly increased after the PA infection (Figure 1C).

### Therapeutic effect of A1AT on lung SPLUNC1, NE activity, bacterial load and inflammation in wild-type mice

**Figure 2:**
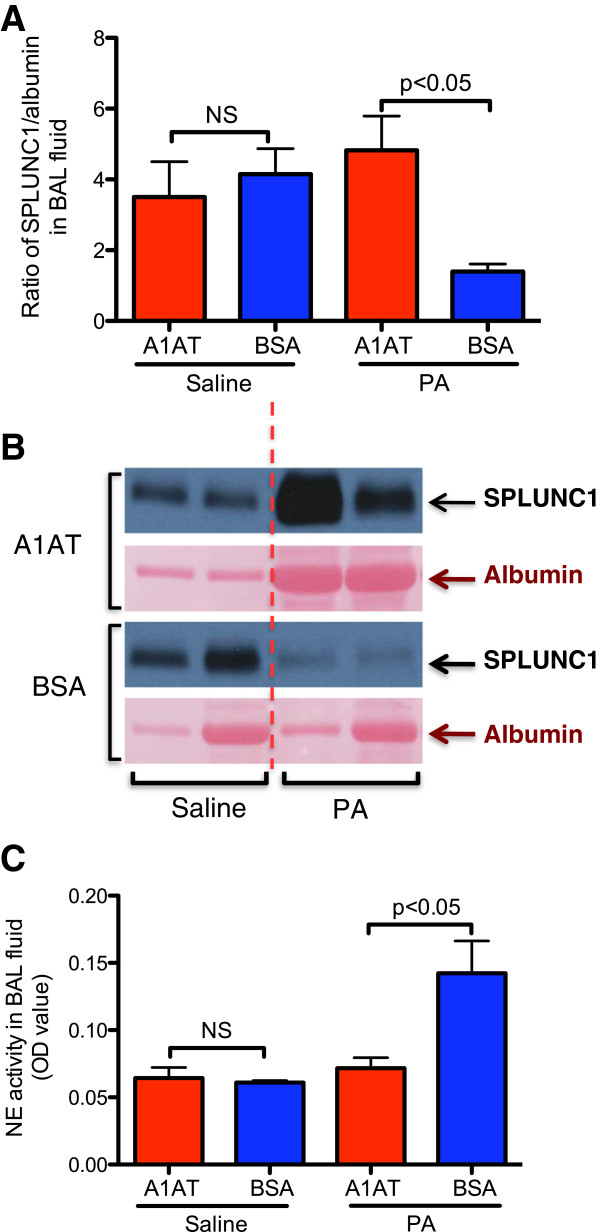
**A1AT treatment enhances SPLUNC1 and reduces NE activity in bronchoalveolar lavage (BAL) fluid of PA-infected WT mice.** PA-infected mice were treated with A1AT for 22 hrs and sacrificed after 24 hrs of infection as described in Materials and Methods. Quantitative analysis **(A)** and representative image **(B)** of BAL fluid SPLUNC1 Western blot were shown to demonstrate the therapeutic effect of A1AT treatment. The two lanes of Western blot image under saline or PA treatment represent SPLUNC1 data from two different mice. NE activity **(C)** was examined by an NE activity assay. N = 4 – 7 mice per group. The vertical dotted red line in Figure 2B separates the saline group from the PA infection group. NS indicates no significant differences. Data are expressed as means ± SEM.

To determine whether A1AT restores SPLUNC1 levels in PA-infected mouse lungs, A1AT was aerosolized to mice 2 hrs after an intranasal inoculation of PA. After 24 hrs of PA infection, A1AT treatment significantly increased SPLUNC1 protein levels in BAL fluid as compared to BSA treatment (Figure 2A and 2B). In line with the SPLUNC1 data, NE activity was reduced following A1AT treatment (Figure 2C).

**Figure 3:**
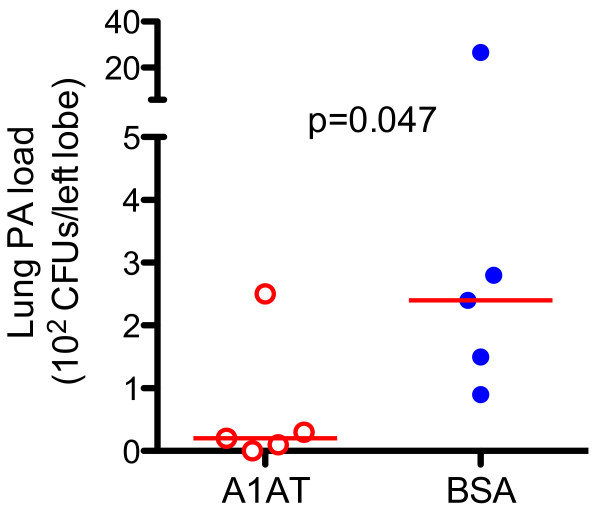
**A1AT treatment reduces lung PA load in wild-type (WT) mice.** Left lungs from PA-infected WT mice were homogenized, and PA load was quantified by culture. The horizontal solid red lines indicate medians of CFUs.

**Figure 4:**
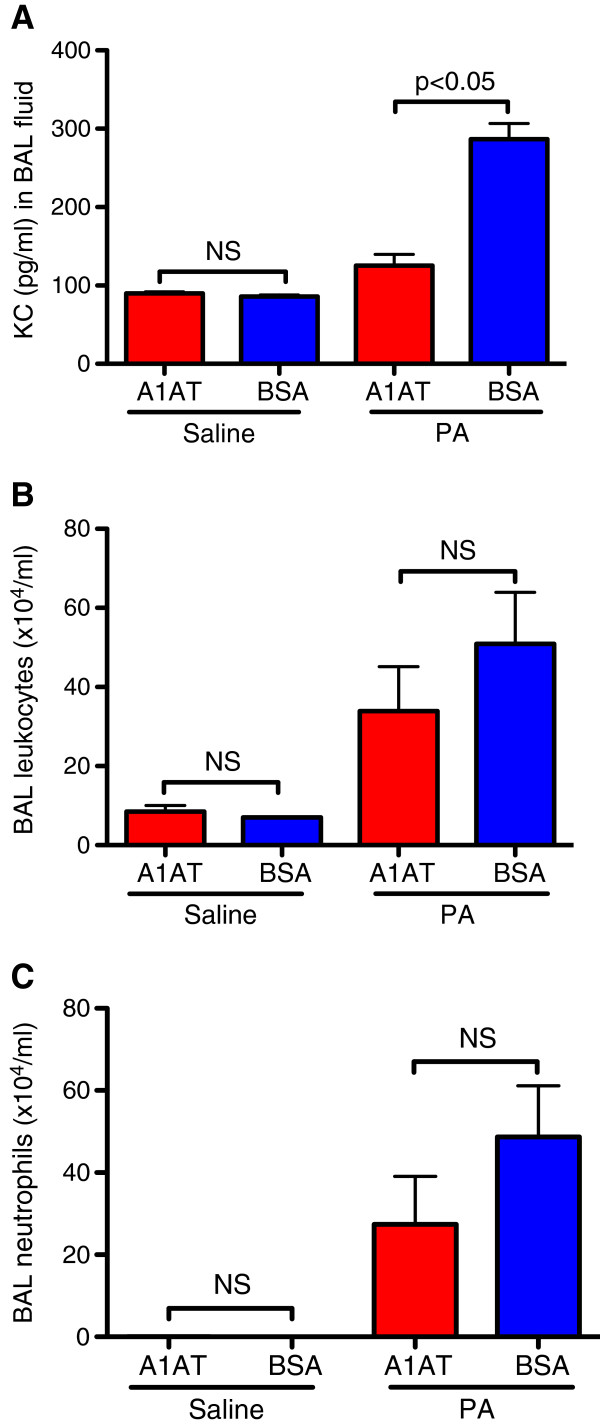
**A1AT treatment reduces PA-induced KC level and trend to reduce total leukocytes in BAL fluid of wild-type (WT) mice. (A)** – KC; **(B)** – total leukocytes; and **(C)** – BAL neutrophils; N = 4 – 7 mice per group. NS indicates no significant differences. Data are expressed as means ± SEM.

Notably, the PA load was reduced by A1AT treatment (Figure 3). Likewise, A1AT significantly reduced PA infection-induced KC (Figure 4A) and trended to reduce total leukocytes (Figure 4B) in BAL fluid. However, the total number of neutrophils in BAL fluid was not significantly reduced by A1AT (Figure 4C).

### No therapeutic effect of A1AT on lung bacterial load and inflammation in SPLUNC1 KO mice

**Figure 5:**
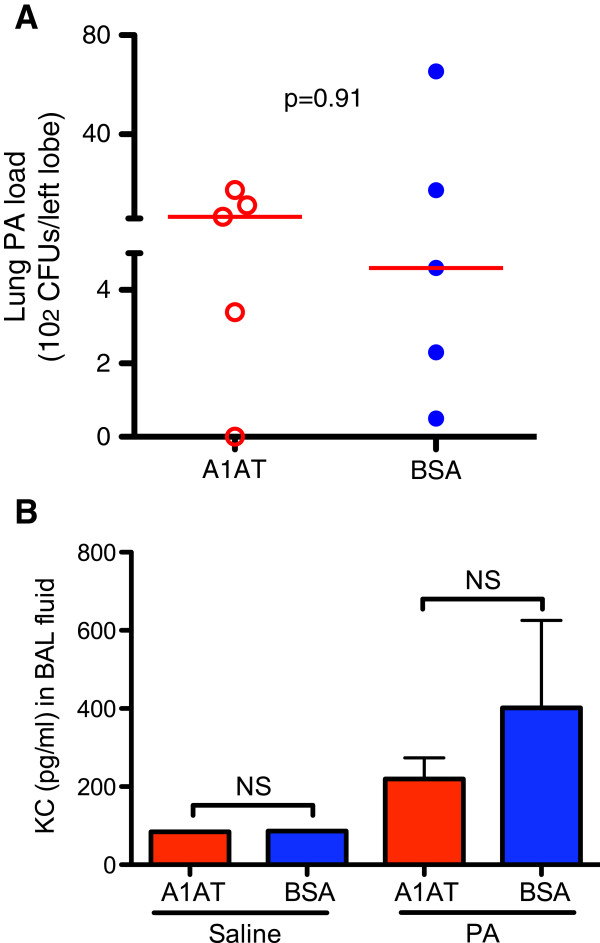
**A1AT treatment does not reduce lung PA load and inflammation in SPLUNC1 knockout (KO) mice. (A)** – PA load was quantified in homogenized left lungs. The horizontal solid red lines indicate medians of CFUs. **(B)** – KC levels were quantified by using an ELISA. N = 4 – 7 mice per group. NS indicates no significant differences. Data are expressed as means ± SEM.

To determine if the therapeutic effect of A1AT is dependent on SPLUNC1, PA-infected SPLUNC1 KO mice were treated with A1AT or BSA. Unlike the WT mice, SPLUNC1 KO mice, following the A1AT treatment, failed to decrease lung PA load and inflammation (Figure 5A and 5B).

## Discussion

The current study provides a novel mechanism by which A1AT exerts host defense function against lung PA infection. By using a SPLUNC1 KO mouse model, we were able to reveal a critical role of SPLUNC1 in A1AT-mediated host defense function. First, PA infection significantly reduced SPLUNC1 expression in wild-type mice, which was accompanied by increased NE activity. Second, A1AT treatment significantly reduced lung PA load and inflammation in wild-type mice, but not in SPLUNC1 KO mice.

Persistent lung PA infection is a significant challenge in the management of CF and COPD patients, particularly during disease acute exacerbations. Activation of airway neutrophils during bacterial infection results in the release of various mediators such as NE that degrades proteins (e.g., elastin, fibronectin), leading to disease progression [7,16]. Although previous studies suggest a therapeutic role of A1AT, an inhibitor of NE, in host defense against bacterial (e.g., PA) infection *in vivo*[6], the underlying mechanisms remain poorly understood. We and others have previously demonstrated that SPLUNC1 exerts host defense function against bacterial infection [7-9], and HNE can degrade secreted SPLUNC1 from airway epithelial cells [7]. We clearly showed that A1AT is effective in reducing PA load in wild-type mice, but not in SPLUNC1 KO mice. Our results suggest that SPLUNC1 is critical to A1AT-mediated host defense function against PA infection.

The impact of an *in vivo* PA infection on SPLUNC1 expression is unclear. Bingle and co-workers have found increased SPLUNC1 protein expression by airway epithelial cells of CF patients [17]. However, this study did not measure the levels of released SPLUNC1 in the airway lumen, and the association of SPLUNC1 levels with NE levels. To clarify the role of an *in vivo* PA infection on SPLUNC1 levels, we examined SPLUNC1 protein in BAL fluid of PA-infected mice. We, for the first time, demonstrated that PA infection reduces SPLUNC1 protein in mouse BAL fluid. The reduced SPLUNC1 may be in part explained by increased NE activity following PA infection as we have shown SPLUNC1 degradation by NE [7]. Additionally, PA-derived elastase may contribute to SPLUNC1 reduction in BAL fluid because it has been shown to degrade some innate immunity components (e.g., surfactant protein A) [18]. We speculate that during PA infection, airway epithelial cells increase intracellular SPLUNC1 expression to compensate the loss of secreted SPLUNC1 due to its degradation by proteases including NE. Although we have shown SPLUNC1 up-regulation by an atypical bacterium *Mycoplasma pneumoniae*[19], the direct effect of PA infection on mouse or human airway epithelial SPLUNC1 expression warrants future study.

One of our major research questions is about whether A1AT can rescue PA infection-induced SPLUNC1 reduction and subsequently enhance host defense against PA infection. To address this question, A1AT was aerosolized to mice 2 hrs after PA infection. This therapeutic approach clearly demonstrated that A1AT treatment restored SPLUNC1 levels in BAL fluid of wild-type mice, which was accompanied by reduced NE activity. Our results have suggested an *in vivo* mechanism of A1AT function. To further demonstrate the role of SPLUNC1 in A1AT therapeutic effect, we treated PA-infected SPLUNC1 KO mice with A1AT. Unlike the wild-type mice, SPLUNC1 KO mice did not show reduced lung PA load. These results emphasize that A1AT therapeutic effect during PA infection depends on SPLUNC1. Given the fact that A1AT can enter into the cells, it would be interesting to determine whether it has a direct effect on intracellular SPLUNC1 mRNA or protein expression. In our primary normal human airway epithelial cell culture experiments, we found that A1AT has no direct effects on intracellular SPLUNC1 expression. Together, A1AT functions primarily through preventing extracellular SPLUNC1 protein from degradation by NE and perhaps other proteases.

We found that lung pro-inflammatory cytokine KC was significantly reduced by A1AT in PA-infected wild-type mouse lungs. However, leukocytes including neutrophils were not as significantly reduced as KC. This may be explained by several factors. First, we only gave one dose of A1AT treatment in PA-infected mice. As a previous study demonstrated a significant inhibition of lung neutrophils following repeated A1AT treatments in cigarette smoke-exposed mice [20], we may need to deliver A1AT multiple times (e.g., before and after PA infection) to markedly suppress lung neutrophil recruitment. Second, A1AT has diverse NE-dependent and NE-independent functions [21]. Lastly, leukocyte numbers may not reflect cell activity. Future studies will explore the direct effects of A1AT on inflammatory cell functions.

**Figure 6:**
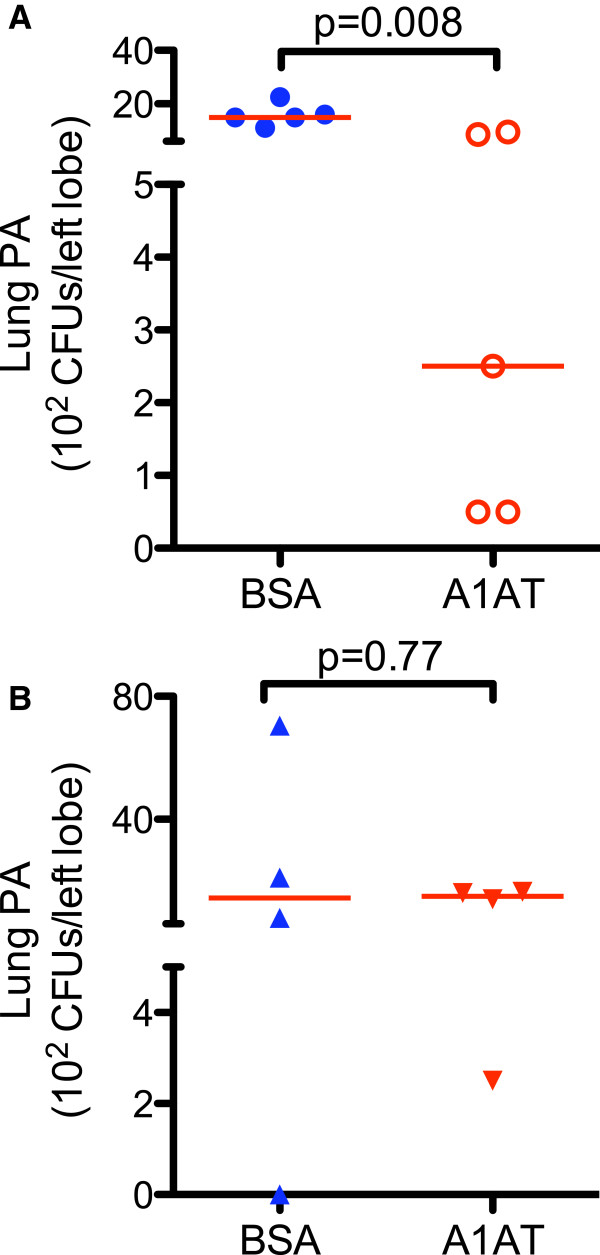
**A1AT pre-treatment reduces lung PA load in wild-type (WT) mice, but not in SPLUNC1 knockout (KO) mice.** WT and SPLUNC1 KO mice were treated with aerosolized A1AT (0.5 mg/ml). Two hours later, mice were intranasally infected with PA (1.5 × 10^7^ CFUs/mouse). After 24 hrs of PA infection, lung tissues were processed to quantify PA load. While A1AT significantly reduced lung PA load in WT mice **(A)**, it did not alter the PA load in SPLUNC1 KO mice **(B)**. N = 4 – 5 mice per group. The horizontal solid red lines indicate medians of CFUs.

We realize that SPLUNC1 KO mice demonstrated greater variability of PA load in the lung than the wild-type mice although the sample size of SPLUNC1 KO and wild-type mice is similar. Thus, it appears that bacterial load variability in our current study is related to the genetic background. We confirmed such greater variability of lung bacterial load in SPLUNC1 KO mice that were pre-treated with A1AT and then infected with PA. We found that A1AT aerosolized 2 hrs prior to PA infection decreased lung PA load in wild-type mice, but not in SPLUNC1 KO mice (Figure 6).

There are several limitations to our current study. First, only one strain of bacteria (i.e., PA) was used in our study. Future studies using other strains of bacteria (e.g., NTHi) are warranted to examine the broad-spectrum effect of A1AT against lung infection. Second, an acute (e.g., 24 hr) PA infection model was carried out to address our research questions. Although we were able to demonstrate a critical role of SPLUNC1 in A1AT-mediated host defense against PA infection with an acute model, a chronic infection model will certainly be beneficial to study the role of SPLUNC1 in disease progression. Lastly, to increase the relevance of our animal research findings to patients, we plan to measure SPLUNC1 and PA in sputum and BAL samples from CF and COPD patients treated with A1AT or placebo.

## Conclusion

Our current study has provided a novel mechanism underlying A1AT-mediated host defense against bacterial infection. Our research findings indicate that A1AT exerts host defense functions against PA infection in part by modulating the host defense protein SPLUNC1.

## Abbreviations

A1AT: α1-antitrypsin; BAL: Bronchoalveolar lavage; CF: Cystic fibrosis; COPD: Chronic obstructive pulmonary disease; KO: Knockout; NE: Neutrophil elastase; NTHi: Non-typeable *Haemophilus influenzae*; PA: *Pseudomonas aeruginosa*; SPLUNC1: Short palate, lung, and nasal epithelium clone 1; WT: Wild-type.

## Competing interest

All authors declare not having competing interests that might have influenced the performance or presentation of our work in this manuscript.

## Authors’ contributions

DJ contributed to the experimental designs, collected the data, carried out data analysis and drafted the manuscript. RP provided the A1AT and revised the manuscript. QW contributed to the data collection, analysis and revised the manuscript. AG contributed to the data collection and revised the manuscript. HWC contributed to the experimental designs, data analysis and critically reviewed the manuscript. All authors read and approved the final manuscript.
